# Serum lncRNAs TUG1, H19, and NEAT1 and their target miR-29b/SLC3A1 axis as possible biomarkers of preeclampsia: Potential clinical insights

**DOI:** 10.1016/j.ncrna.2024.06.007

**Published:** 2024-06-08

**Authors:** Mahmoud A. Senousy, Olfat G. Shaker, Ahmed H.Z. Elmaasrawy, Ahmed M. Ashour, Shuruq E. Alsufyani, Hany H. Arab, Ghada Ayeldeen

**Affiliations:** a Department of Biochemistry, Faculty of Pharmacy, Cairo University, Cairo, 11562, Egypt; b Department of Medical Biochemistry and Molecular Biology, Faculty of Medicine, Cairo University, Cairo, Egypt; c Department of Obstetrics and Gynecology, Faculty of Medicine, Zagazig University, Zagazig, Egypt; d Department of Pharmacology and Toxicology, College of Pharmacy, Umm Al Qura University, P.O. Box 13578, Makkah, 21955, Saudi Arabia; e Department of Pharmacology and Toxicology, College of Pharmacy, Taif University, P.O. Box 11099, Taif, 21944, Saudi Arabia

**Keywords:** Cystine transporter, lncRNAs, microRNAs, Preeclampsia, SLC3A1

## Abstract

To date, the epigenetic signature of preeclampsia (PE) is not completely deciphered. Oxidative stress-responsive long non-coding RNAs (lncRNAs) are deregulated in preeclamptic placenta; however, their circulating profiles and diagnostic abilities are still unexplored. We investigated serum redox-sensitive lncRNAs TUG1, H19, and NEAT1, and their target miR-29b/cystine/neutral/dibasic amino acids transporter solute carrier family 3, member 1 (SLC3A1) as potential non-invasive biomarkers of PE risk, onset, and severity. We recruited 82 patients with PE and 78 healthy pregnant women. We classified PE patients into early-onset (EOPE) and late-onset (LOPE) subgroups at a cut-off 34 gestational weeks and into severe and mild PE subgroups by blood pressure and proteinuria criteria. Bioinformatics analysis was employed to select lncRNAs/microRNA/target gene interactions. Serum H19, NEAT1, and SLC3A1 mRNA expression were reduced, meanwhile miR-29b levels were elevated, whereas there was no significant difference in TUG1 levels between PE patients and healthy pregnancies. Serum H19 levels were lower, whereas miR-29b levels were higher in EOPE versus LOPE. Serum miR-29b and H19 levels were higher in severe versus mild PE. ROC analysis identified serum H19, NEAT1, miR-29b, and SLC3A1 as potential diagnostic markers, with H19 (AUC = 0.818, 95%CI = 0.744–0.894) and miR-29b (AUC = 0.82, 95%CI = 0.755–0.885) were superior discriminators. Only H19 and miR-29b discriminated EOPE and severe PE cases. In multivariate logistic analysis, miR-29b and H19 were associated with EOPE, using maternal age and gestational age as covariates, while miR-29b was associated with severe PE, using maternal age as covariate. Studied markers were correlated with clinical and ultrasound data in the overall PE group. Serum H19 and TUG1 were negatively correlated with albuminuria in EOPE and LOPE, respectively. NEAT1 and SLC3A1 were correlated with ultrasound data in EOPE. Likewise, TUG1, miR-29b, and SLC3A1 showed significant correlations with ultrasound data in LOPE. Conclusively, this study configures SLC3A1 expression as a novel potential serum biomarker of PE and advocates serum H19 and miR-29b as biomarkers of EOPE and miR-29b as a biomarker of PE severity.

## Highlights

• To our knowledge, serum SLC3A1 expression and correlation with ultrasound data in PE is first evidenced.
• Serum H19, NEAT1, miR-29b, and SLC3A1 could have utility as new biomarkers in the diagnosis of PE.
• Serum H19 and miR-29b may be associated with early-onset PE.
• Serum miR-29b may be associated with severe PE.
• SLC3A1 correlated negatively with miR-29b and positively with TUG1, H19, and NEAT1.

**Table:** List of Abbreviations

Abbreviation	Full name
ACOG	American College of Obstetricians and Gynecologists
ALT	Alanine transaminase
AF	Amniotic fluid
AIM2	Absent in melanoma 2
ARE	Antioxidant response element
ARHGAP26	Rho GTPase activating protein 26
AUC	Area under the curve
BMI	Body mass index
ceRNA	Competing endogenous RNA
CRP	C-reactive protein
CS	Caesarean section
DBP	Diastolic blood pressure
dMSC	Decidua-derived mesenchymal stem cell
EOPE	Early-onset preeclampsia
EVT	Extravillous trophoplast
FBW	Fetal birth weight
GA	Gestational age
GSH	Reduced glutathione
H19	Imprinted maternally expressed transcript
HAT	Heterodimeric amino acid transporter
HUVEC	Human umbilical vein endothelial cell
INR	International normalized ratio
IUGR	Intrauterine growth restriction
lncRNAs	Long non-coding RNAs
LOPE	Late-onset preeclampsia
MAP	Mean arterial pressure
MCL-1	Myeloid cell leukemia-1
miRNA	MicroRNA
MMP	Matrix metalloproteinase
MOD	Mode of delivery
MPP^+^	1-methyl-4-phenylpyridinium
NEAT1	Nuclear-enriched abundant transcript 1
NPV	Negative predictive value
Nrf2	Nuclear factor erythroid 2-related factor 2
Par6	Par-6 partitioning defective 6 homolog gamma
PE	Preeclampsia
PI	Pulsatility index
PPV	Positive predictive value
RhoA	Ras homolog family member A
RND3	Rho family GTPase 3
ROS	Reactive oxygen species
ROC	Receiver-operating characteristic
RT	Reverse transcription
RT-qPCR	Reverse transcriptase-quantitative polymerase chain reaction
SBP	Systolic blood pressure
SLC3A1	Solute carrier family 3, member 1
Smurf1	Smad ubiquitination regulatory factor1
SN	Sensitivity
SP	Specificity
TβR3	Type III transforming growth factor-β receptor
TLC	Total leukocyte count
TUG1	Taurine-upregulated gene 1
VEGFA	Vascular endothelial growth factor A
VD	Vaginal delivery

## 1 Introduction

After 20 weeks of pregnancy, preeclampsia (PE) develops as a clinical multi-system disease with several manifestations, including new-onset hypertension, proteinuria, and other forms of impaired end-organ function [1]. Worldwide, PE affects an estimated 4 million women annually, and without proper and timely intervention, PE causes adverse maternal and fetal outcomes, and over 70,000 maternal deaths and 500,000 fetal deaths globally every year [2].

Although PE is diagnosed by blood pressure criteria and proteinuria, new effective biomarkers are clinically imperative to identify at-risk pregnant women at early stage, avoid misdiagnosis of true patients with PE according to the clinical diagnostic criteria, ensure timely delivery, reduce maternal and perinatal morbidity and mortality, prognosticate PE severity, and provide novel targets for therapy [3]. Although there are advances in prediction and prevention of preterm PE (<37 weeks), prediction of term (≥37 weeks) and postpartum PE is limited with no preventive treatments [2]. This necessitates a newfangled understanding of the molecular underpinnings of PE pathogenesis which may furnish new early diagnostic markers and future therapeutic targets.

Placental oxidative stress is one of the hallmarks in the pathogenesis of PE and is tightly connected to its onset [4]. Early-onset PE (EOPE; <34 weeks) occurs due to impaired placentation in early pregnancy and features as shallow invasion of placental trophoblast cells with subsequent endothelial dysfunction and poor spiral artery remodeling, culminating in insufficient blood flow and oxygenation to the placenta and enhanced oxidative stress [5]. Late-onset PE (LOPE; ≥34 weeks) is a condition characterized by the overcrowding of villous tissues at term. This condition is partially triggered by oxidative changes in the placenta, resulting in impaired trophoblast cell growth [5]. As a result of placental oxidative stress, excessive production of cytokines and anti-angiogenic signals occurs, and downstream long non-coding RNAs (lncRNAs) are generated [6,7].

Compelling evidence has supported a strong connection between lncRNAs and oxidative stress [7,8]. Noteworthy, several lncRNAs have been documented to exert a role in the setting of oxidative stress and are linked to the oxidation/antioxidant system, in particular the nuclear factor erythroid 2-related factor 2 (Nrf2)/antioxidant response element (ARE) axis [7]. In addition, lncRNAs act as competing endogenous RNAs (ceRNAs) for microRNAs (miRNAs) involved in regulating the oxidative stress-related genes [7]. Indeed, some lncRNAs have been functionally implemented in oxidative stress related-diseases and emerged as specific biomarkers empowered by their tissue-specific features [[8], [9], [10], [11]]. Recently, aberrantly expressed lncRNAs in placental tissues and blood of PE patients have been elaborated in relation with the pathophysiological processes of PE, including trophoblast invasion and migration, angiogenesis, inflammation, and oxidative stress [6,12,13]. Moreover, the potential clinical utility of circulating lncRNAs as non-invasive biomarkers of PE has been largely expatiated [[14], [15], [16], [17]]. However, the exact role of redox-sensitive lncRNAs in PE pathogenesis, their diagnostic ability, and their potential as therapeutic targets remain to be clarified, necessitating more research in the field.

Accordingly, a bioinformatics analysis has been conducted in this study to identify redox-sensitive lncRNAs related to PE and to pinpoint a common miRNA/target gene for them. Three lncRNAs taurine-upregulated gene 1 (TUG1), imprinted maternally expressed transcript (H19), and nuclear-enriched abundant transcript 1 (NEAT1) and their common target miR-29b/solute carrier family 3, member 1 (SLC3A1) axis were selected as possible biomarkers.

TUG1 is a redox-sensitive lncRNA that regulates oxidative stress and apoptosis via the miR-144/Nrf2 axis [18]. Overexpression of TUG1 promoted cell proliferation, migration, invasion, and angiogenesis, and activated vascular endothelial growth factor A (VEGFA) in hypoxia-reoxygenation-stimulated human umbilical vein endothelial cells (HUVECs) by acting as a miR-29a sponge [19]. H19, another redox-sensitive lncRNA, has been shown to be sensitive to H_2_O_2_ treatment [11] and to regulate the antioxidant function in cardiac progenitor cells [20], cholangiocarcinoma cells [21], and diabetic mouse model by targeting miR-657 [22]. H19 is highly expressed in early placental trophoblasts particularly extravillous trophoplasts (EVT) to promote cell invasion via targeting miR-106a/VEGFA1 axis, indicating the role of H19 in early placentation [23]. NEAT1 is another oxidative stress-responsive lincRNA. NEAT1 knockdown prevented MPP^+^-induced inflammatory response, oxidative stress, and apoptosis in dopaminergic neuroblastoma cells via miR-1277-5p/ARHGAP26 axis [24], on the other hand its high expression counteracted the H_2_O_2_-induced neuronal damage in neuro2A cells [25]. In PE, NEAT1 silencing promoted Treg/Th17 balance by modulating the miR-485-5p/AIM2 axis [26]. Interestingly, these selected lncRNAs act as ceRNAs via commonly sponging miR-29b in different models [[27], [28], [29]].

miR-29b regulates oxidative stress and angiogenesis in different diseases, including PE [19,30]. A novel predicted target of miR-29b is the cystine transporter SLC3A1; this interaction was revealed in cystinuria patients [31]. SLC3A1 gene is located at 2p21 and encodes for the SLC3A1 protein, also known as rBAT, a type II membrane glycoprotein and an essential component of the plasma membrane heterodimeric amino acid transporter (HAT) known as rBAT/b^0/+^AT, which is responsible for the transport of cystine and neutral and dibasic amino acids in a Na^+^-independent manner. This HAT consists of a heavy chain (SLC3) and a light chain (SLC7). SLC3A1 is one of two identified heavy chains, SLC3A1 and SLC3A2, essential for the localization in plasma membrane and the stabilization of light chain (SLC7A9), thus SLC3A1/SLC7A9 combination forms the functional HAT [32]. The SLC3A1 protein is mainly expressed in the apical membrane of the renal proximal tubules and intestinal mucosal cells [33], but also important to the nutrient transport capacity in the placenta [34]. SLC3A1 gene mutations and deletions were associated with cystinuria [32]. SLC3A1 is also needed for cysteine transport, which with glycine and glutamate, is important in the synthesis of reduced glutathione (GSH), an important cellular antioxidant [35]. Intriguingly, SLC3A1 increased the cysteine uptake and hence GSH synthesis, and reduced reactive oxygen species in breast cancer cells [35]. Howbeit, the possible role of SLC3A1 and its crosstalk with miR-29b and the upstream lncRNAs TUG1, H19, and NEAT1 in PE and their diagnostic potential remain largely unknown.

In this context, we hypothesized that TUG1, H19, NEAT1, miR-29b, along with SLC3A1 could have aberrant expression in the oxidative stress and inflammatory milieu of PE and could be leaked to the bloodstream from damaged placenta. Thereby, the current study appraised the expression profiles of serum TUG1, H19, NEAT1, and their predicted target miR-29b/SLC3A1 axis as potential biomarkers of PE risk, onset, and severity. We also investigated the correlation between these parameters and maternal, fetal, and ultrasound data in patients with PE.

## 2 Subjects and methods

### 2.1 Patients

One hundred and sixty Egyptian pregnant women were recruited in this prospective study from the maternity wards of the Department of Obstetrics and Gynecology, Zagazig University as well as Kasr Al-Ainy hospital, Cairo University where they received routine obstetric examination. Eighty-two pregnant women with PE participated in the study, while seventy-eight age-matched healthy pregnant women served as the control group.

Upon admission, all participants were subjected to full history gathering, routine physical and clinical examination, and laboratory investigations. Patients’ data, including information on pregnancy history, risk factors, and perinatal outcome were registered in the medical records and analyzed in this study.

The control group comprised healthy pregnant females with normal blood pressure and ultrasound data and without proteinuria or any complications. The diagnosis of PE was set before blood sampling upon the criteria of new-onset hypertension (systolic blood pressure (SBP) = 140 mmHg or higher and/or diastolic blood pressure (DBP) = 90 mmHg or higher on at least two occasions 4 h apart after 20 weeks of pregnancy in combination with new-onset proteinuria (1+ or higher urine dipstick testing of two random urine samples collected at least 4 h apart) as described in the guidelines of the American College of Obstetricians and Gynecologists (ACOG 2020) [36].

According to PE onset, patients were categorized as EOPE and LOPE by 34 weeks. Patients developing clinical manifestations of PE and requiring delivery before 34 weeks of gestation were assigned as EOPE, whereas patients were regarded as LOPE if occurred at or after 34 gestational weeks [37]. Thirty-nine percent of PE patients were EOPE (n = 32/82), while the remaining cases were LOPE.

The severity of PE was determined according to the guidelines of ACOG 2020 [36]. Patients were assigned with severe PE when having SBP = 160 mmHg or higher and/or DBP = 110 mmHg or higher on two different occasions, combined with proteinuria (urine dipstick 2+ or higher) amalgamated by the presence of other complications. A mild case was defined as having SBP between 140 and 159 mmHg or DBP between 90 and 109 mmHg in two separate incidents as well as 1+ proteinuria on a dipstick test. Among the recruited PE patients, 28/82 cases (34 %) were diagnosed with mild PE, while the remaining cases were having severe PE.

At admission, pregnant females were routinely subjected to blood pressure measurement and ultrasound investigation, and then blood samples were collected from patients diagnosed with PE and from normal pregnant women as well. The pregnancy weeks (gestational age, GA), amniotic fluid (AF) status, abnormal Doppler, and the presence of intrauterine growth restriction (IUGR) were determined at admission by ultrasonography using an ultrasound device for fetal measurements and before blood sampling. Abnormal Doppler was judged using uterine artery Doppler (considered abnormal when mean pulsatility index (PI) above 95th centile) and/or umbilical artery Doppler (considered abnormal in case of absent or reversed end diastolic flow or PI above 95th centile). IUGR was diagnosed based on estimated fetus weight below the 3rd centile or below the 10th centile for the GA with evidence of placental dysfunction either abnormal uterine artery Doppler and/or abnormal umbilical artery Doppler. To note, body mass index (BMI) was determined by measuring the body weight and height (kg/m^2^) of each participant at the time of inclusion. After delivery, we recorded the fetal birth weight (FBW) for clinical correlations.

The inclusion criteria for the present study were pregnant females from 20 to 40 weeks of gestation who were not subjected to any invasive procedure. The exclusion criteria were women who had pre-existing hypertension, gestational diabetes, twin pregnancy, intrauterine fetal death, or hemostatic abnormalities, cancer, cardiovascular, metabolic, autoimmune, renal, and hepatic diseases.

All procedures were performed in accordance with the guidelines and regulations of World Medical Association (Helsinki declaration). All the recruited PE patients and controls signed a formal written informed consent before participating in the study and all experiments were done after approval of the ethical committee of the Faculty of Pharmacy, Cairo University, Cairo, Egypt (Permit number: BC3130).

### 2.2 Bioinformatics analysis to select lncRNAs/miRNA/target gene axis

#### 2.2.1 Selection of PE-related lncRNAs

Selection of PE-associated lncRNAs was done using the LncRNA and Disease Database (version 2.0) (LncRNADisease v2.0) (http://www.rnanut.net/lncrnadisease/), a new database developed to screen for disease-associated lncRNAs and circular RNAs [38]. We searched the database using the disease name “Pre-eclampsia”. The output was 126 non-coding RNAs. The highly-scored PE-associated lncRNAs, along with their detection method (experimental/predicted) and scores are listed in Supplementary Table S1. We then filtered this output based on criteria of score >0.75 (Table S1) and/or having mechanistic link to PE, having biological and experimental links to redox status, oxidative stress, or oxidative stress-related diseases, and its clinical relevance to PE is unknown or not sufficiently elucidated. TUG1, HOTAIR, MALAT1, and H19 were among the highly-ranked results that fulfilled these criteria; however, we studied the clinical relevance of serum MALAT1 and HOTAIR in a previous study [14]. NEAT1 was recently connected to PE in vitro [26], and there is a gap of knowledge regarding its clinical impact. Finally, TUG1, H19, and NEAT1 were selected based on their link to oxidative stress [11,18,[20], [21], [22],^24^,^25^] and that their mechanistic links to PE were experimentally validated [19,23,26,39,40].

#### 2.2.2 Prediction of lncRNA-miRNA and miRNA-target gene interactions

Using the transcriptome-wide miRNA target predictions from the miRcode 11 database (http://www.mircode.org/), the miRNA targets of the selected lncRNAs were compiled. The predictions were made based on the GENCODE transcripts. According to this database, the miR-29 family was a common target for the three selected lncRNAs (TUG1, H19, and NEAT1). Afterwards, we examined whether the selected lncRNAs interacted specifically with miR-29b in an experimental setting [[27], [28], [29]].

According to the Human microRNA Disease Database v3.2 (https://www.cuilab.cn/hmdd), miR-29b is associated with PE and has previously been experimentally validated in a previous study [41]. Out of multiple targets of miR-29b in public databases, we have chosen SLC3A1 as a novel oxidative stress-related target. In order to verify the interaction between miR-29b and SLC3A1, TargetScan 7.0 database (https://www.targetscan.org/vert_80/) and miRcode 11 database were used. This interaction was also validated [31,42].

### 2.3 Blood collection and serum separation for RNA assay

The study involved the collection of approximately 5 mL of venous blood from each participant using a plain tube following a vein puncture. Serum was prepared within 30 min after blood collection by leaving the blood to clot at room temperature, then centrifuging at 2000 g for 15 min. Hemolysis- and sediment-free supernatant was quickly taken out, fractionated into aliquots, and frozen at −80 °C until further use.

### 2.4 RNA analysis

#### 2.4.1 RNA extraction

To eliminate traces of cellular debris and red blood cells, serum samples were centrifuged at 3000 g for 5 min before RNA extraction. Total RNA extraction from serum (200 μl) was conducted using Qiagen miRNeasy Serum/Plasma kit, as instructed by the vendor. Extracted RNA yield and purity were measured by a nanodrop (Bioanalyzer Agilent RNA 6000 picoassay). Samples with A260/280 ratio between 1.8 and 2.2 were further used for reverse transcription (RT).

#### 2.4.2 Assay for lncRNAs and SLC3A1 mRNA expression using reverse transcriptase-quantitative polymerase chain reaction (RT-qPCR)

RT was attempted on 100 ng of total RNA using the high capacity cDNA Reverse Transcriptase kit (Applied Biosystems, USA) in a 20 μl RT reaction mixtures following the manufacturer's protocol. The RT runs were conducted using the following thermal cycler conditions: 10 min at 25 °C, 110 min at 37 °C, and 5 s at 95 °C. Using GAPDH as an internal control gene, the expression profiles of TUG1, H19, NEAT1, and SLC3A1 were determined by qPCR. The GAPDH gene has been validated previously for use as an excellent internal control for normalizing lncRNAs [43,44]. The qPCR assay was conducted using the Maxima SYBR Green PCR kit (ThermoFischer, USA) and customized primers (Metabion, Germany) following the manufacturer's recommendations. The sequences of primers used are shown in Table 1. The NCBI Primer-BLAST tool (https://www.ncbi.nlm.nih.gov/tools/primer-blast/) was used to confirm the specificity of primers. Melting curve analysis was also performed to assure the absence of primer dimers. Using the Rotorgene Q system (Qiagen), real-time PCR was implemented in 20 μl reaction mixtures with applying thermal cycling conditions as follows: 95 °C for 10 min, followed by 40 cycles at 95 °C for 15 s and 60 °C for 60 s.

**Table 1:** Customized primers’ sequences used in qPCR.

Gene	Primer sequence (5′–3′)
TUG1	**F:** TAGCAGTTCCCCAATCCTTG
	**R:** CACAAATTCCCATCATTCC
H19	**F:** ATCGGTGCCTCAGCGTTCGG
	**R:** CTGTCCTCGCCGTCACACCG
NEAT1	**F:** TGGCTAGCTCAGGGCTTCAG
	**R:** TCTCCTTGCCAAGCTTCCTTC
SLC3A1	F: TTATGTCTFCGGAGTGCCTTC
	R: CACCAGCACAGAAGCCACTG
GAPDH	**F:** GAAGGTCGGAGTCAACGGATT
	**R:** CGCTCCTGGAAGATGGTGAT

#### 2.4.3 miR-29b assay using RT-qPCR

The miScript II RT kit (Qiagen) was used for reverse-transcribing 0.1 μg of total RNA in a 20 μl final volume following the instructions of the manufacturer. The RT was executed using the thermal parameters: 60 min at 37 °C and 5 min at 95 °C. For qPCR, the miScript SYBR Green PCR kit (Qiagen) was employed to prepare a total of 20 μl reaction mixtures, as guided by the vendor. To this end, we mixed the appropriate volume of the cDNA with the ready-made miScript reverse (Universal) primer and specific forward primers for hsa-miR-29b-3p and SNORD68 (internal control). Our research group [45,46] and other reports [47] have previously validated SNORD68 as an internal control for miRNA normalization. As SNORD68 expression is stable and consistent across both ovarian cancer cells and normal cells, it can be used as a reliable reference for estimating relative miRNA levels [47]. We employed the Rotorgene Q real-time system (Qiagen) with the following PCR thermal conditions: 15 min at 95 °C, 40 cycles of 15 s at 94 °C followed by 30 s at 55 °C and 30 s at 70 °C. The fold change was calculated for the expression of studied genes using the formula 2^-ΔΔCt^, where ΔΔCt = ΔCt_patient_ - ΔCt_control group_.

### 2.5 Statistical analysis

Statistical analysis was conducted using SPSS (version 15, SPSS, Chicago, IL) and GraphPad Prism (version 7.0, GraphPad Software, CA, USA). Values are shown as mean ± standard deviation (SD), median (25 %–75 % percentiles), or number (percentage) when appropriate. To compare categorical data, Fisher's exact test or chi-square test was used. In order to determine the normality of continuous variables, the Shapiro Wilk and Klomogrov Simirnov tests were conducted. One-way ANOVA or Kruskal Wallis tests were used to compare data from three independent groups when appropriate. For comparisons between two independent groups, the Student's *t*-test or Mann-Whitney *U* test was used. The receiver-operating characteristic (ROC) analysis was applied to determine the diagnostic accuracy of molecular data. Among the discriminators, we considered the area under the curve (AUC) = 0.6 to 0.69 as a significant discriminator, AUC = 0.7–0.89 as a potential discriminator, and AUC = 0.9 or higher as an excellent discriminator. In this study, we present the sensitivity, specificity, positive predictive value (PPV), and negative predictive value (NPV) of each molecular marker at the best cut-off value determined by the fold change at which the sum of sensitivity and specificity is maximized. For establishing the association between biomarkers, early PE risk, and PE progression risk, we used univariate logistic analysis, followed by a stepwise multivariate logistic regression analysis controlled with confounders as maternal age and GA. Spearman rank coefficient was used to identify the correlation between parameters. For all tests, statistical significance was set at a two-tailed *P* < 0.05.

The G*Power software version 3.1.9.7 was applied to estimate the initial sample size based on the following assumptions: two independent groups (PE cases versus controls), effect size = 0.5 (fold change 1.25 in PE cases versus 1 in control), population variance (SD = 0.5), case/control ratio = 1, type I error α = 0.05, and type II error β = 0.2. It has been determined that a minimum total sample size of 128 (64 in cases and 64 in controls) would yield a two-tailed power of 0.8. Additionally, these assumptions led to a two-tailed power of 85 % based on a total sample size of 160 (82 + 78) in this study.

## 3 Results

### 3.1 Characteristics of EOPE and LOPE patients

Table 2 displays the demographic, anthropometric, biochemical, and ultrasound data of EOPE and LOPE patients, along with fetal characteristics such as GA and FBW. Smoking was more frequent in both EOPE and LOPE patients than in healthy pregnancies (*P* = 0.046). Notably, 68.75 % of EOPE cases were diagnosed with severe PE as reflected in the observed higher frequency of dipstick 4+ albuminuria in EOPE than that in LOPE cases. In contrast, dipstick 1+ albuminuria was more frequent in LOPE than in EOPE (*P* = 0.008). EOPE patients showed higher C-reactive protein (CRP) levels than those in the control group, but no statistical difference was observed between LOPE and control pregnancies. LOPE patients exhibited significantly higher serum creatinine and uric acid levels than levels in the control pregnancies. Both EOPE and LOPE patients demonstrated more frequent IUGR, abnormal Doppler, low AF, and cesarean delivery (*P* < 0.0001 for each) than in the control group; however, FBW was significantly lower in EOPE patients than LOPE patients or control pregnancies (*P* < 0.0001). To note, EOPE and LOPE patients had comparable maternal age, BMI, blood pressure, hematological parameters, glycemic status, liver function tests, and ultrasound data (*P* > 0.05).

**Table 2:** Characteristics of EOPE, LOPE patients, and healthy pregnancies.

Parameters	Controls (n = 78)	EOPE (n = 32)	LOPE (n = 50)	*P-*value
Maternal age (years), Range	31.0 ± 6.6 (18.0–41.0)	32.1 ± 6.8 (19.0–42.0)	28.2 ± 6.6 (18.0–41.0)	0.13
Body mass index (BMI) (kg/ $m^{2}$)	32.4 ± 4.7	30.9 ± 5.5	31.4 ± 3.3	0.57
Smoking, n (%)				0.046*
Yes	27 (34.6)	16 (50.0)	28 (56.0)	
No	51 (65.4)	16 (50.0)	22 (44.0)	
SBP (mmHg)	116.8 ± 12.9^A^	164.1 ± 20.1^B^	164.6 ± 16.2^B^	<0.0001*
DBP (mmHg)	72.3 ± 7.0^A^	109.7 ± 14.8^B^	107.4 ± 8.0^B^	<0.0001*
MAP (mmHg)	94.6 ± 8.3^A^	136.9 ± 16.2^B^	136.0 ± 10.8^B^	<0.0001*
CRP (mg/L)	6.0 (3.0–30.0)^A^	37.0 (4.3–110.0)^B^	24 (8.4–31.5)^AB^	0.012*
Albuminuria, n (%)				0.008*
4+	Nil	12 (37.5)	6 (12.0)	
3+		8 (25.0)	14 (28.0)	
2+		10 (31.25)	14 (28.0)	
1+		2 (6.25)	16 (32.0)	
Hb (g/dL)	10.7 ± 1.3	10.7 ± 1.3	10.7 ± 1.2	0.99
TLC *1000 (cells/mm^3^)	8.1 ± 2.6	8.3 ± 3.2	8.2 ± 3.0	0.98
Platelet count *1000 (cells/mm^3^)	304.3 ± 80.8	290.2 ± 99.8	284.6 ± 94.8	0.67
Fasting plasma glucose (mg/dL)	82.9 ± 13.8	81.2 ± 17.0	81.3 ± 11.2	0.87
2 h-postprandial plasma glucose (mg/dL)	115.8 ± 14.0	121.4 ± 17.7	116.3 ± 13.4	0.412
ALT (U/L)	18.4 ± 10.1	18.3 ± 8.3	22.2 ± 8.9	0.24
Albumin (g/dL)	3.19 ± 0.27	3.18 ± 0.23	3.08 ± 0.45	0.419
Total bilirubin (mg/dL)	0.60 ± 0.25	0.54 ± 0.29	0.53 ± 0.24	0.507
INR	0.96 ± 0.07	0.95 ± 0.08	0.948 ± 0.07	0.7
Creatinine (mg/dL)	0.7 ± 0.2^A^	0.8 ± 0.2^AB^	0.83 ± 0.27^B^	0.029*
Uric acid (mg/dL)	3.6 ± 0.4^A^	3.8 ± 0.5^AB^	4.0 ± 0.8^B^	0.026*
GA (weeks)	37.6 ± 1.5^A^	30.1 ± 1.2^B^	38.0 ± 1.1^A^	<0.0001*
FBW (kg)	3.28 ± 0.38^A^	2.53 ± 0.53^B^	3.04 ± 0.35^A^	<0.0001*
IUGR, n (%)				<0.0001*
No	72 (92.3)	16 (50.0)	26 (52.0)	
Yes	6 (7.7)	16 (50.0)	24 (48.0)	
Doppler, n (%)				<0.0001*
Normal	72 (92.3)	16 (50.0)	32 (64.0)	
Abnormal	6 (7.7)	16 (50.0)	18 (36.0)	
Amniotic fluid, n (%)				<0.0001*
Normal	72 (92.3)	8 (25.0)	28 (56.0)	
Low	6 (7.7)	24 (75.0)	22 (44.0)	
MOD, n (%)				0.0047*
VD	31 (40.0)	4 (12.5)	10 (20.0)	
CS	47 (60.0)	28 (87.5)	40 (80.0)	
PE Severity, n (%)	–			0.81
Mild		10 (31.25)	18 (36.0)	
Severe		22 (68.75)	32 (64.0)	

Results are presented as mean ± SD, median (25 %-75 % percentiles) or number (percentage). Groups with different letters are statistically significant, whereas groups with the same letters are non-significant. Chi-square or Fisher's exact tests were used for categorical data. Continuous parameters were compared using one-way ANOVA followed by Tukey's post hos test, except CRP data were compared using Kruskal Wallis test followed by Dunn's multiple comparison test. * indicates statistical significance, *P* < 0.05. ALT, alanine transaminase; BMI, body mass index; CRP, C-reactive protein; CS, Caesarean section; DBP, diastolic blood pressure; EOPE, early-onset PE; FBW, fetal birth weight; GA, gestational age; Hb, hemoglobin; INR; international normalized ratio; IUGR, intrauterine growth restriction; LOPE, late-onset preeclampsia; MAP, mean arterial pressure; MOD, mode of delivery; PE, preeclampsia; SBP, systolic blood pressure; TLC, total leukocyte count; VD, vaginal delivery.

### 3.2 Results of bioinformatics analysis

**Fig. 1:**
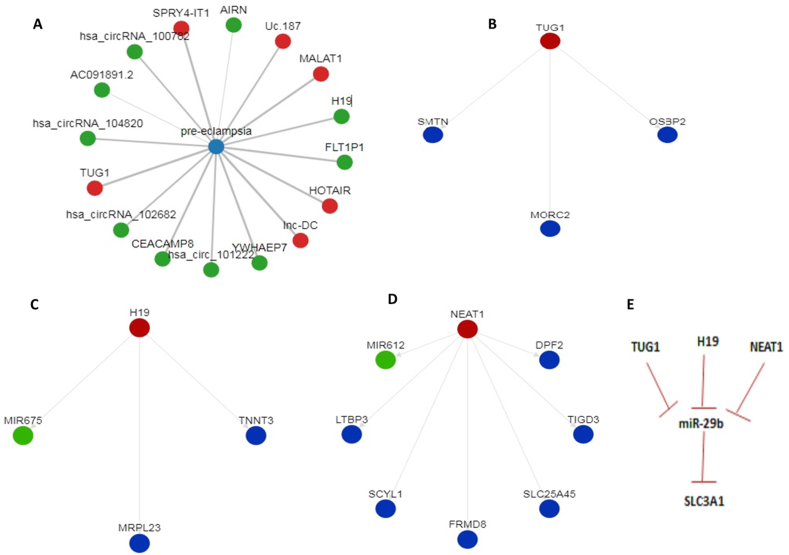
**Highly-scored PE-related non-coding RNAs and target genes of TUG1, H19, and NEAT1 related to PE according to the LncRNADisease v2.0.** A: represents highly-scored PE-related non-coding RNAs. B, C, D: present the target genes of selected lncRNAs, TUG1, H19, and NEAT1, respectively as reported in the LncRNADisease v2.0. E presents the results of our target prediction analysis that miR-29b is a common target of TUG1, H19, and NEAT1 as verified in the miRcode 11 database, and that SLC3A1 is a miR-29b target as reported in TargetScan 7.0 database and verified in the miRcode 11 database.

The highly-ranked non-coding RNAs associated with PE according to the LncRNADisease v2.0, including lncRNAs H19 and TUG1 are displayed in Fig. 1A. Fig. 1B–D illustrates the target genes of H19, TUG1, and NEAT1 reported in the LncRNADisease v2.0 in relation to PE. Fig. 1E presents the results of our target prediction analysis conducted in this study. The interactions of TUG1, H19, and NEAT1 transcripts with miR-29abcd family were verified in the miRcode 11 database and are listed in Supplementary Table S2. The predicted interaction between miR-29b-3p and human SLC3A1 mRNA was recorded in the TargetScan 7.0 database (Supplementary Table S3) and verified in the miRcode 11 database (Supplementary Table S2).

### 3.3 Serum H19, NEAT1, miR-29b, and SLC3A1 are differentially expressed in the sera of PE patients

**Fig. 2:**
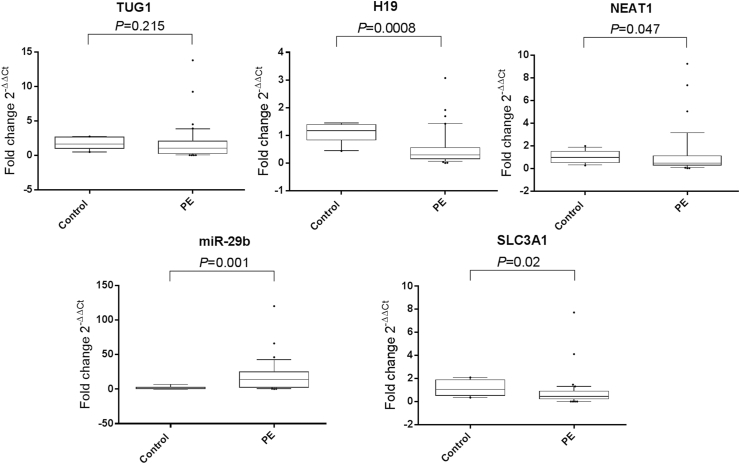
**Serum expression profiles of TUG1, H19, NEAT1, miR-29b,and SLC3A1 in PE patients.** The box represents the 25 %–75 % percentiles; the line inside the box represents the median and the upper and lower lines representing the 10 %–90 % percentiles of the fold change of studied parameters in PE patients (n = 82) compared to healthy pregnancies (n = 78). Data were analyzed using Mann-Whitney *U* test. *P* < 0.05 denotes statistical significance. The dots represent the outliers. The median (25 %–75 % percentiles) values of the fold change are TUG1 1.064 (0.26–2.099), H19 0.35 (0.16–0.65), NEAT1 0.50 (0.32–1.14), miR-29b 14.12 (2.79–25.02) and SLC3A1 0.44 (0.23–0.94) in PE patients normalized to healthy pregnancies. The figure shows downregulation of H19, NEAT1, and SLC3A1 by 2.85, 2, 2.27-fold, respectively, and upregulation of miR-29b by 14.12-fold in the sera of PE patients versus healthy pregnancies.

As depicted from Fig. 2, serum H19, NEAT1 lncRNAs, and SLC3A1 mRNA expression levels showed 2.85, 2, 2.27-fold downregulation (*P* = 0.0008, 0.047, 0.02, respectively), whereas serum miR-29b expression levels were upregulated by 14.12-fold (*P* = 0.001) in PE patients compared with levels in the healthy pregnancies. Meanwhile, there was no significant difference in serum TUG1 expression between patients with PE and healthy pregnancies (*P* = 0.215).

### 3.4 Serum H19 and miR-29b are associated with the onset and severity of PE

**Fig. 3:**
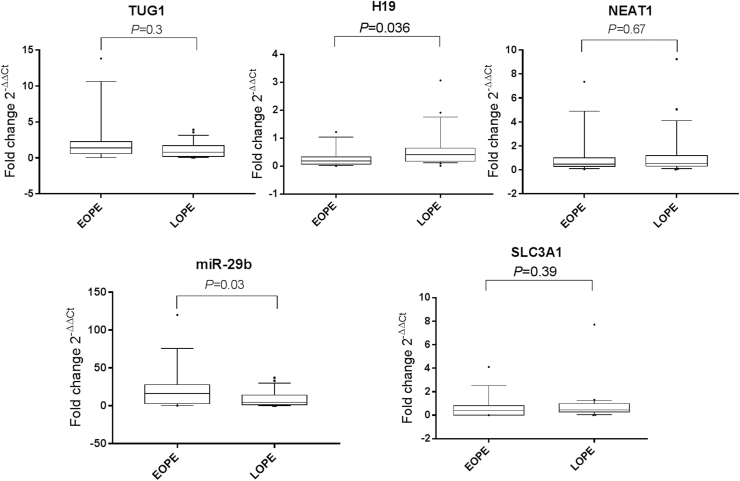
**Serum expression profiles of TUG1, H19, NEAT1, miR-29b,and SLC3A1 in EOPE and LOPE patients.** The box represents the 25 %–75 % percentiles; the line inside the box represents the median and the upper and lower lines representing the 10 %–90 % percentiles of the fold change of studied parameters in EOPE patients (n = 32) compared with that in LOPE patients (n = 50). Fold change (2^−ΔΔCt^) in EOPE and LOPE groups was calculated relative to their corresponding early (n = 40) and late control pregnancies (n = 38) matched with gestational weeks. Data were analyzed using Mann-Whitney *U* test. *P* < 0.05 denotes statistical significance. The median values of the fold change are TUG1 (1.41 vs 0.81), H19 (0.2 vs 0.4), NEAT1 (0.48 vs 0.54), miR-29b (16.45 vs 6.84) and SLC3A1 (0.43 vs 0.46) in EOPE versus LOPE, respectively. The figure shows that serum H19 levels were lower by 2-fold, whereas miR-29b levels were higher by 2.4-fold in EOPE versus LOPE.

To contemplate the association of studied parameters with PE onset, we compared their expression levels between EOPE and LOPE cases. Only H19 and miR-29b levels figured out to be differentially expressed between EOPE and LOPE patients (Fig. 3). Notably, serum H19 expression levels were lower (*P* = 0.036), whereas miR-29b levels were higher in EOPE versus LOPE cases (*P* = 0.03). Serum TUG1, NEAT1, and SLC3A1 levels were not significantly different in this comparison (*P* = 0.3, 0.67, and 0.39, respectively).

**Fig. 4:**
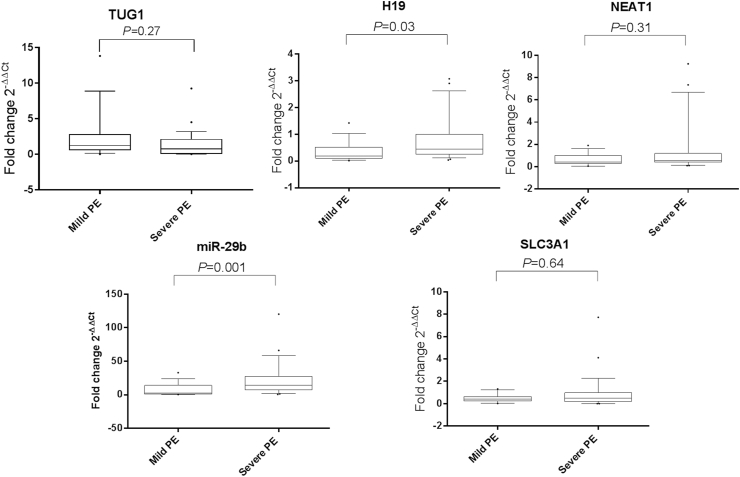
**Serum expression profiles of TUG1, H19, NEAT1, miR-29b and SLC3A1 in mild and severe PE patients.** The box represents the 25 %–75 % percentiles; the line inside the box represents the median and the upper and lower lines representing the 10 %–90 % percentiles of the fold change of studied parameters in mild PE cases (n = 28) compared with that in severe PE cases (n = 54). Data were analyzed using Mann-Whitney *U* test. *P* < 0.05 denotes statistical significance. The median values of the fold change are TUG1 (1.27 vs 0.77), H19 (0.19 vs 0.45), NEAT1 (0.42 vs 0.52), miR-29b (2.71 vs 14.22), and SLC3A1 (0.41 vs 0.57) in mild versus severe PE, respectively. The figure shows that serum H19 and miR-29b levels were higher in severe versus mild PE by 2.36 and 5.2-fold, respectively.

When dichotomizing PE cases into mild and severe PE, we found that serum levels of H19 and miR-29b were higher in patients with severe PE compared with levels in patients having mild PE (*P* = 0.03 and 0.001, respectively) (Fig. 4). Again, no significant changes were recorded in the serum levels of TUG1 and NEAT1 lncRNAs as well as SLC3A1 mRNA expression between the two groups (*P* = 0.27, 0.31, and 0.64, respectively).

### 3.5 Significant correlations are noticed between studied molecular markers in the whole PE, EOPE, and LOPE groups

No significant correlations were observed between studied molecular markers in the healthy control pregnancies (Supplementary Table S4); however, multiple significant correlations were recorded between them in the whole PE group (Table 3). Serum expression levels of SLC3A1 mRNA were positively correlated with the levels of TUG1 (r = 0.417, *P* < 0.0001), H19 (r = 0.491, *P* < 0.0001), and NEAT1 (r = 0.417, *P* = 0.0003), while demonstrated a strong negative correlation with miR-29b levels (r = −0.708, *P* < 0.0001) in the whole PE group. Notably, the correlations of SLC3A1 with NEAT1 (r = 0.432, *P* = 0.028), H19 (r = 0.476, *P* = 0.006), and miR-29b (r = −0.726, *P* < 0.001) remained and was intensified with TUG1 (r = 0.726, *P* < 0.0001) in the EOPE group.

**Table 3:** Correlation of studied molecular parameters with each other in PE patients.

Data	TUG1	H19	NEAT1	miR-29b	SLC3A1
TUG1					
r	–	0.210	0.171	−0.282	0.417
*P*		0.057	0.157	0.022^a^	<0.0001^a^
H19					
r	0.210	–	0.614	0.092	0.491
*P*	0.057		<0.0001^a^	0.462	<0.0001^a^
NEAT1					
r	0.171	0.614	–	0.063	0.417
*p*	0.157	<0.0001^a^		0.637	0.0003^a^
miR-29b					
r	−0.282	0.092	0.063	–	−0.708
*P*	0.022^a^	0.462	0.637		<0.0001^a^
SLC3A1					
r	0.417	0.491	0.417	−0.708	–
*P*	<0.0001^a^	<0.0001^a^	0.0003^a^	<0.0001^a^	

Correlations were done using Spearman correlation in the whole PE group (n = 82).

a *P* < 0.05: statistically significant.

Serum TUG1 levels showed a negative correlation with miR-29b levels (r = −0.282, *P* = 0.022) in the total PE group which was intensified in EOPE group (r = −0.431, *P* = 0.017). Serum TUG1 was positively correlated with H19 in EOPE group (r = 0.519, *P* = 0.002), but not in the whole or LOPE groups (*P* > 0.05).

Serum H19 and NEAT1 levels were strongly correlated in the same direction (r = 0.614, *P* < 0.0001) in the whole group and the correlation was intensified in separate LOPE group (r = 0.769, *P* < 0.0001).

### 3.6 Serum levels of studied molecular markers are correlated with clinicopathological data in the whole PE, EOPE, and LOPE groups

In healthy pregnancies, serum H19 was correlated with DBP (r = 0.529, *P* = 0.016) (Supplementary Table S4). Interestingly, serum levels of the examined parameters in the whole recruited preeclamptic women (Table 4) as well as in EOPE and LOPE subgroups showed multiple significant correlations with the clinicopathological parameters. In overall PE patients, serum TUG1 was negatively correlated with the presence of IUGR (r = −0.237, *P* = 0.032), while showed positive correlations with maternal age (r = 0.303, *P* = 0.006), BMI (r = 0.307, *P* = 0.005), and FBW (r = 0.26, *P* = 0.018). Serum H19 was inversely correlated with the presence of low AF (r = −0.299, *P* = 0.006), while was positively correlated with platelet count (r = 0.250, *P* = 0.025), SBP (r = 0.27, *P* = 0.014), DBP (r = 0.296, *P* = 0.007), and mean arterial pressure (MAP) (r = 0.308, *P* = 0.005). Serum NEAT1 recorded a positive correlation with platelet count (r = 0.298, *P* = 0.012) and negative correlations with the presence of abnormal Doppler (r = −0.322, *P* = 0.007) and low AF (r = −0.246, *P* = 0.049). Serum miR-29b was inversely correlated with GA (r = −0.245, *P* = 0.048), while showed positive correlations with the presence of abnormal Doppler (r = 0.271, *P* = 0.028) and cesarean delivery (r = 0.417, *P* = 0.0004). Serum SLC3A1 recorded a positive correlation with FBW (r = 0.283, *P* = 0.01) and negative correlations with the presence of IUGR (r = −0.32, *P* = 0.003), abnormal Doppler (r = −0.421, *P* < 0.0001), low AF (r = −0.355, *P* = 0.001), and cesarean delivery (r = −0.245, *P* = 0.049).

**Table 4:** Correlations of studied molecular parameters with maternal, fetal, and ultrasound data in PE patients.

Data	TUG1	H19	NEAT1	miR-29b	SLC3A1
Maternal age					
r	0.303	0.108	0.021	0.043	0.191
*P*	0.006*	0.336	0.863	0.734	0.080
BMI					
r	0.307	0.170	0.173	−0.135	0.024
*P*	0.005*	0.128	0.151	0.280	0.830
Smoking					
r	0.142	−0.058	0.221	0.183	0.205
*P*	0.204	0.605	0.066	0.142	0.064
SBP					
r	0.008	0.270	0.015	−0.054	0.024
*P*	0.947	0.014*	0.904	0.666	0.830
DBP					
r	−0.044	0.296	0.105	−0.097	0.048
*P*	0.695	0.007*	0.386	0.437	0.670
MAP					
r	0.005	0.308	0.039	−0.051	0.035
*P*	0.964	0.005*	0.749	0.681	0.750
CRP					
r	0.036	−0.058	−0.117	0.053	−0.054
*P*	0.749	0.749	0.334	0.675	0.630
Albuminuria					
r	−0.042	0.155	0.099	0.149	0.018
*P*	0.708	0.164	0.413	0.233	0.870
Platelet count					
r	0.187	0.250	0.298	−0.146	0.122
*p*	0.093	0.025*	0.012*	0.242	0.270
GA					
r	**−**0.059	0.090	0.035	−0.245	0.074
*P*	0.510	0.423	0.773	0.048*	0.510
FBW					
r	0.260	0.029	0.016	0.063	0.283
*P*	0.018*	0.795	0.898	0.602	0.010*
IUGR					
r	−0.237	0.025	−0.210	−0.174	−0.320
*P*	0.032*	0.825	0.081	0.164	0.003*
Abnormal Doppler					
r	−0.111	−0.134	−0.322	0.271	−0.421
*P*	0.321	0.230	0.007*	0.028*	<0.0001*
Low AF					
r	−0.135	−0.299	−0.246	−0.232	−0.355
*P*	0.227	0.006*	0.049*	0.060	0.001*
MOD					
r	−0.074	0.077	−0.074	0.417	−0.245
*P*	0.509	0.493	0.541	0.0004*	0.049*

Correlations were done using Spearman correlation in the whole PE group (n = 82). *P < 0.05: statistically significant. AF, amniotic fluid; BMI, body mass index; CRP, C-reactive protein; DBP, diastolic blood pressure; FBW, fetal birth weight; GA, gestational age; IUGR, intrauterine growth restriction; MAP, mean arterial pressure; MOD, mode of delivery; SBP, systolic blood pressure.

In EOPE patients, serum H19 was negatively correlated with the extent of albuminuria (r = − 0.464, *P* = 0.007). Serum NEAT1 recorded a negative correlation with the presence of IUGR (r = −0.557, *P* = 0.003) and a positive correlation with FBW (r = 0.482, *P* = 0.013). Serum SLC3A1 mRNA expression was correlated with abnormal Doppler (r = −0.424, *P* = 0.016) and its correlation with the presence of IUGR (r = −0.407, *P* = 0.021) was intensified.

In LOPE patients, serum TUG1 was negatively correlated with the extent of albuminuria (r = − 0.386, *P* = 0.006) and the presence of low AF (r = −0.346, *P* = 0.014) and its correlations with IUGR and FBW (r = 0.48, *P* = 0.0004) were intensified. Serum miR-29b recorded a positive correlation with the presence of low AF (r = 0.405, *P* = 0.008), while SLC3A1 mRNA expression was positively correlated with FBW (r = 0.287, *P* = 0.043) and its correlation with the presence of abnormal Doppler (r = −0.555, *P* < 0.0001) and low AF (r = −0.425, *P* = 0.002) were intensified. Altogether, these correlations might implement the aberrantly expressed levels of studied parameters with the clinical setting of EOPE and LOPE, especially the ultrasound data.

### 3.7 Serum H19, NEAT1, miR-29b, and SLC3A1 have diagnostic potential in PE

**Fig. 5:**
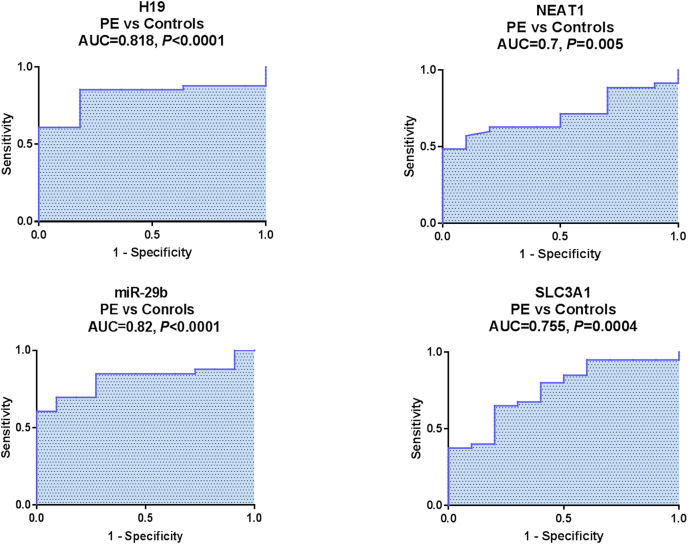
**Diagnostic performance of serum H19, NEAT1, miR-29b,and SLC3A1 in PE.** An analysis of the ROC curves for the studied parameters to distinguish between PE group (n = 82) and healthy pregnancies (n = 78). *P* < 0.05 denotes statistical significance. ROC analysis revealed serum H19, NEAT1, miR-29b, and SLC3A1 as potential diagnostic markers, with H19 (AUC = 0.818, 95%CI = 0.744–0.894) and miR-29b (AUC = 0.82, 95%CI = 0.755–0.885) are superior to other markers in PE diagnosis.

In the ROC curve analysis, serum H19, NEAT1, miR-29b, and SLC3A1 expression levels were potential discriminators of PE patients from the healthy control pregnancies with AUCs = 0.818, 0.7, 0.82, and 0.755, respectively (*P* < 0.05) (Fig. 5). By comparison, H19 and miR-29b have comparable AUCs and were superior to NEAT1 and SLC3A1. The sensitivities, specificities, PPV, and NPV at the best cut-off values (at which the sum of sensitivity and specificity is maximum) are presented in Table 5.

**Table 5:** Diagnostic performance of studied biomarkers.

Parameter	AUC (95 % CI)	*P*	Best cutoff (fold)	SN (%)	SP (%)	PPV (%)	NPV (%)
**PE vs healthy controls**							
H19	0.818 (0.744–0.894)	<0.0001*	<0.70	85.4	81.8	83.3	84.2
NEAT1	0.700 (0.619–0.781)	0.005*	<0.61	57.1	90.0	81.4	59.8
miR-29b	0.820 (0.755–0.885)	<0.0001*	>2.00	69.7	90.9	89.1	74.0
SLC3A1	0.755 (0.680–0.830)	0.0004*	<0.58	65.0	80.0	77.9	68.5
**EOPE vs LOPE**							
H19	0.695 (0.575–0.815)	0.003*	<0.28	75.0	72.0	63.2	81.8
miR-29b	0.710 (0.592–0.828)	0.002*	>14.17	73.3	78.3	67.6	81.3
**Severe vs mild PE**							
H19	0.650 (0.592–0.771)	0.023*	>0.29	70.4	71.4	84.4	56.8
miR-29b	0.738 (0.631–0.845)	0.001*	>5.49	95.0	66.7	72.9	86.4

The best cutoff value was selected as the fold change at which the sum of sensitivity and specificity is maximum. *P < 0.05: statistically significant. SN, sensitivity; SP, specificity; PPV, positive predictive value; NPV, negative predictive value. PE, preeclampsia, n = 82; healthy controls, n = 78; EOPE, early-onset PE, n = 32; LOPE, late-onset PE, n = 50; mild PE, n = 28; severe PE, n = 54.

Among studied parameters, serum H19 recorded the highest sensitivity (85.4 %) and NPV (84.2 %) which could exclude the presence of PE, whereas miR-29b showed the highest specificity (90.9 %) and PPV (89.1 %) which could support the diagnosis of PE.

### 3.8 Serum H19 and miR-29b discriminate PE cases according to onset and severity

**Fig. 6:**
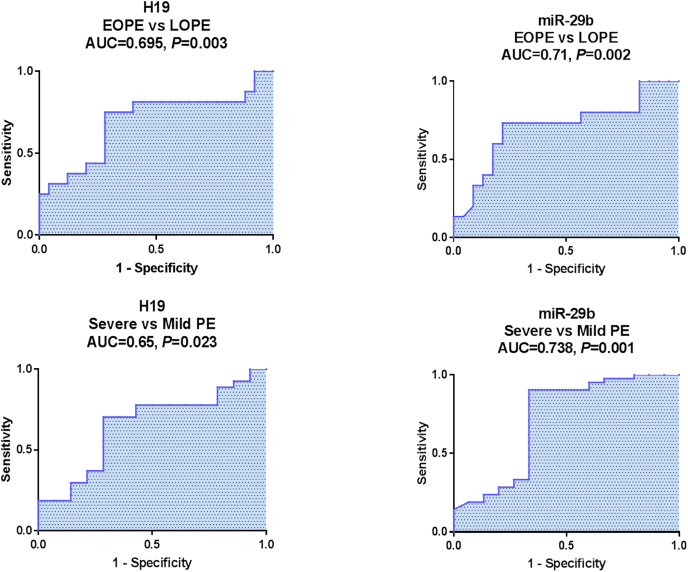
**Performance of serum H19 and miR-29b to distinguish early and severe PE cases.** ROC curve analysis to discriminate EOPE (n = 32) versus LOPE patients (n = 50) and severe (n = 54) versus mild PE patients (n = 28). *P* < 0.05 denotes statistical significance. ROC analysis identified that H19 and miR-29b distinguished EOPE from LOPE cases and severe from mild PE cases with moderate accuracy.

Serum H19 and miR-29b discriminated EOPE from LOPE patients with AUCs = 0.695 and 0.71, respectively (*P* < 0.05) and also distinguished severe PE cases from mild cases with AUCs = 0.65 and 0.738, respectively in the ROC curve analysis (Fig. 6). The calculated sensitivities, specificities, positive and negative predictive values at the best cut-off values (at maximum sum of sensitivity and specificity) are displayed in Table 5.

In discriminating EOPE from LOPE, serum H19 recorded higher NPV (81.8 %) which could exclude the presence of EOPE, whereas miR-29b showed higher PPV (81.3 %) which help early detection of PE.

Regarding PE severity, serum H19 recorded higher PPV (84.4 %) which could help to confirm the presence of severe PE, whereas miR-29b showed higher sensitivity (95 %) and NPV (86.4 %) which could confirm the diagnosis of severe PE.

### 3.9 Serum H19 and miR-29b are associated with EOPE and miR-29b is associated with severe PE in logistic regression analysis

Using univariate and multivariate logistic regression analyses, we uncovered the variables associated with the risk of EOPE (EOPE vs early control pregnancies) (Table 6) and severe PE (severe vs mild PE) (Table 7). Along with blood pressure, CRP, and ultrasound data, serum H19 and miR-29b appeared as negative and positive variables associated with EOPE, respectively in the univariate analysis (*P* < 0.05). With adjustment by maternal age and GA as confounders, serum miR-29b and SBP configured as the final independent positive variables, whereas H19 was a negative independent variable associated with EOPE in the multivariate analysis (Table 6).

**Table 6:** Association of serum H19 and miR-29b with EOPE compared with early controls using logistic regression analysis.

Parameter	Beta coefficient	SE (%)	*P*	OR	95 % CI
**Univariate Analysis**					
H19	−1.714	23.3	0.015*	0.18	0.05–0.76
miR-29b	0.198	40.4	0.012*	1.22	1.09–2.98
SBP	0.470	34.0	0.004*	1.60	1.16–2.19
DBP	0.708	49.4	0.044*	2.03	1.02–3.96
MAP	0.392	35.7	0.0035*	1.48	1.14–1.93
CRP	0.039	33.3	0.0008*	1.04	1.02–1.07
IUGR	1.946	34.8	0.0007*	7.00	2.06–19.83
Abnormal Doppler	2.197	31.0	0.0002*	9.00	2.74–26.98
Low amniotic fluid	4.140	21.7	<0.0001*	63.00	13.7–239.4
**Multivariate Analysis**^a^					
H19	−1.770	27.7	0.025*	0.17	0.052–0.77
miR-29b	0.536	48.5	0.020*	1.71	1.1–2.88
SBP	0.565	38.9	0.005*	1.76	1.62–2.13
Constant	−8.990				

Univariate analysis was done using early-onset PE cases, n = 32 cases and early healthy pregnancies, n = 40 matched with gestational weeks at delivery (*P* > 0.05). Significant variables were then entered into stepwise forward multivariate analysis (*P* < 0.05 for entering and *P* < 0.1 for removal from the model). - 2 log likelihood of the model*, P* < 0.0001. Standard error (SE) is presented as percentage of the beta coefficient. *Statistically significant, *P* < 0.05. CI, confidence interval; CRP, C-reactive protein; DBP, diastolic blood pressure; FBW, fetal birth weight; IUGR, intrauterine growth restriction; MAP, mean arterial pressure; OR, odds ratio; SBP, systolic blood pressure. ^a^ controlled by maternal age and gestational age as covariates.

**Table 7:** Association of serum miR-29b with severe PE using logistic regression analysis.

Parameter	Beta coefficient	SE (%)	*P*	OR	95 % CI
**Univariate Analysis**					
H19	0.049	30.6	0.030*	1.05	1.007–3.66
miR-29b	0.095	31.5	0.01*	1.10	1.02–4.69
GA	−0.094	180.9	0.590	0.91	0.654–1.271
SBP	0.104	31.7	0.002*	1.11	1.039–1.18
DBP	0.290	32.0	0.002*	1.34	1.11–1.601
MAP	0.198	31.3	0.0015*	1.22	1.08–1.38
CRP	0.002	450.0	0.770	1.002	0.986–1.02
Creatinine	3.770	43.8	0.023*	43.42	1.69–110.7
Uric acid	1.990	43.5	0.021*	7.37	1.349–40.23
IUGR	2.480	35.1	0.004*	12.00	2.198–65.51
Abnormal Doppler	2.940	37.8	0.008*	18.90	2.15–66.27
Low amniotic fluid	0.980	69.4	0.223	2.67	0.608–8.447
**Multivariate Analysis**^**a**^					
H19	0.005	40.0	0.100	1.005	0.92–3.68
miR-29b	0.077	28.6	0.020*	1.08	1.01–4.50
SBP	0.140	42.9	0.008*	1.15	1.05–1.18
Constant	−32.150				

Univariate analysis was done using mild PE, n = 28; severe PE, n = 54 cases. Significant variables were then entered into stepwise forward multivariate analysis (*P* < 0.05 for entering and *P* < 0.1 for removal from the model). - 2 log likelihood of the model*, P* < 0.0001. Standard error (SE) is presented as percentage of the beta coefficient. *Statistically significant, *P* < 0.05. CI, confidence interval; CRP, C-reactive protein; DBP, diastolic blood pressure; GA, gestational age; IUGR, intrauterine growth restriction; MAP, mean arterial pressure; SBP, systolic blood pressure. ^a^ controlled by maternal age as covariate.

A univariate analysis unveiled miR-29b to be associated with PE severity, along with blood pressure, ultrasound data, serum creatinine, and uric acid levels (*P* < 0.05). Evaluating the performance of these parameters in the multivariate analysis unraveled miR-29b and SBP as the final independent variables associated with PE severity after adjusting with maternal age (Table 7).

## 4 Discussion

The search for circulating biomarkers associated with EOPE and severe PE is ongoing for targeted surveillance. In PE, a large number of placental-specific exosomes is released into the circulation due to endothelial dysfunction; these exosomes encapsulate RNA, DNA, and proteins [48]. This provides a mean by which several lncRNA, miRNAs, and mRNAs, including SLC3A1 mRNA to be present in the circulation. Here, we attested the clinical value of the serum levels of redox-sensitive lncRNAs H19 and NEAT1, along with their predicted target miR-29b/SLC3A1 axis.

To our knowledge, this is the first report that configured the clinical correlation of this regulatory network especially SLC3A1 with maternal and fetal parameters in PE. As we hypothesized, the present results figured out differential expression of serum H19, NEAT1, miR-29b, and SLC3A1 in the sera of PE patients; howbeit opposing to our hypothesis, we failed to find significant alterations of serum TUG1 levels compared to their healthy pregnancy counterparts. Notably, we noticed a decline of serum H19, NEAT1, and SLC3A1, along with escalated expression levels of miR-29b in PE patients. Furthermore, we spotlighted the association of serum H19 and miR-29b with EOPE, and serum miR-29b with PE severity and progression.

The downregulation of serum H19 in PE patients was similar to other reports [[49], [50], [51]]. In contrast to our findings, a higher placental tissue H19 has been reported in PE patients than healthy controls [52,53]. However, several underlying mechanisms exist to explain our finding. Downregulated H19 decreases transforming growth factor-β signaling via the Par6/Smurf1/RhoA pathway activated by TβR3, leading to impaired migration and invasion of EVT cells [51]. H19 was also reported to have antioxidant function in several models [[20], [21], [22]] that could be exhausted in the oxidative stress milieu of PE. In addition, the presence of rs2107425 polymorphism in H19 gene has been linked to a higher PE risk [53].

Similar to our findings, differential expression of H19 in EOPE and severe PE patients was also noticeable. H19 was among 32 differentially expressed lncRNAs and there was a downregulation of its expression in term-placenta of patients with early-onset severe PE [54]. H19 expression level was lower in EOPE placentas than in normal placentas [49], and its derived miR-675-5p inhibited nodal modulator 1 which attenuated the proliferation of trophoblasts [49]. In addition, a microarray analysis showed lowered levels of H19 in the villous tissues from idiopathic recurrent miscarriage patients [23]. Herein, the negative correlation of H19 with the extent of albuminuria in EOPE patients advocates its relation with PE development.

These findings are also supported by other studies that evaluated H19 levels in early placentation. It seems likely that H19, which has a hypoxia-resposive region in the promoter of its gene, is normally highly expressed in early placenta, where hypoxia is evident, to support EVT invasion, then the levels of H19 decreases thereafter [55]. In human first-trimester EVT cell lines, overexpression of H19 promoted cell migration and tube formation via competing with miR-106a-5p, thus elevated VEGF to increase angiogenesis [23]. H19 also supported early EVT invasion via increased expression of marix metalloproteinases MMP13 and MMP14 [39]. Interestingely, H19 has been shown to be upregulated in blood samples at early gestation in pregnant women who later developed PE [55]. Together, these findings suggest circulating maternal H19 as a marker of EOPE.

Similar to H19, we observed decreased levels of NEAT1 in the sera of PE patients; however, its levels were not differentially expressed between EOPE vs LOPE or mild vs severe PE patients. This finding could be explained based on that NEAT1 showed antioxidant functions by counteracting superoxide and H_2_O_2_ [24,25], thus its levels could be reduced during oxidative stress. Conversely, the expression of NEAT1 was markedly higher in placental samples of PE than control rats [56]. NEAT1 overexpression halted trophoblast cell proliferation, migration, invasion, and colony formation, but enhanced cell apoptosis; this was accomplished via sponging miR-373 and regulating Fms-like tyrosine kinase-1, suggesting that NEAT1 could modulate PE development [56]. Moreover, NEAT1 was upregulated in PE and its knockdown beneficially modulated the miR-485-5p/AIM2 axis in trophoblast cells to promote Treg/Th17 immune balance [26]. In EOPE patients, we recorded a negative correlation between NEAT1 and IUGR. Howbeit; this result is contradictory to the previously reported data [57,58]. The placentas of fetal-growth restricted fetus have been shown to express NEAT1 at higher levels, causing increased paraspeckles in villous trophoblasts and retention of mRNAs within their nuclei [57,58].

In the current study, miR-29b was found to be upregulated in the PE patients’ sera and higher levels were associated with EOPE and severe PE. In agreement with these results, elevated expression of miR-29b-3p was noticed in maternal plasma at the time of severe PE compared with that in time-matched controls [41]. In severe PE patients, miR-29b overexpression was reported in decidua-derived mesenchymal stem cells (dMSCs). Notably, miR-29b overexpression targeted histone deacetylase 4 to dampen dMSCs proliferation and HUVECs migration/tubule-formation abilities [59]. Mechanistically, miR-29b induces apoptosis and inhibits invasion and angiogenesis of trophoblast cells via targeting MMP2/MCL1/VEGFA [60], suggesting its relation to PE development and severity.

The upregulated levels of miR-29b could also be attributed to its role in promoting oxidative stress [30]. Additionally, miR-29b has been reported to be sponged by H19 and NEAT1 [27,28], thus the downregulation of these lncRNAs in this study could presumably escalate the miR-29b levels. The correlations of miR-29b with the ultrasound data especially in overall PE and LOPE patients accentuate its involvement in PE pathology.

To the best of our knowledge, this study is the first to provide evidence about differential expression of serum SLC3A1, its diagnostic potential, and correlations with maternal and fetal parameters in PE. Intriguingly, the potential role of SLC3A1 in cancer was identified in breast cancer. SLC3A1 expression has been shown to be upregulated in breast cancer cell lines, increased cysteine uptake, and promoted tumorigenesis of breast cancer cells [35]. SLC3A1 was also found to be highly expressed in breast cancer tissues compared to peritumoral tissues and was correlated with breast cancer histological grade and progression [35]. Howbeit; we demonstrated serum SLC3A1 downregulation in PE. This could be due to overexpression of upstream miR-29b as SLC3A1 is reported as a predicted miR-29b target in TargetScan database, and this interaction was evidenced in cystinuria patients [31]. In addition, the decline in SLC3A1 could presumably evidences the decrease in cysteine uptake in the placenta and hence reduction in *de novo* GSH synthesis leading to exacerbation of the oxidative stress state. This hypothesis could be advocated by the observed correlations between serum SLC3A1 and miR-29b (negative correlation) and H19, TUG1, and NEAT1 (positive correlations) which were also obvious in EOPE subgroup. In addition, the recorded positive correlations of SLC3A1 with ultrasound data in overall PE patients were intense in EOPE or LOPE patients; this preludes its link to PE development and onset. These data suggest the potential role of SLC3A1 in PE and provides H19/miR-29b/SLC3A1 or NEAT1/miR-29b/SLC3A1 axes as new perspective in PE development and pathology and as potential targets for intervention. These axes need further cellular investigation.

In opposition to our hypothesis, the lncRNA TUG1 levels were not altered in the sera of PE patients. In contrast, another report demonstrated TUG1 downregulation in the placentas of PE patients, which curbed the invasion and migration of trophoblast-like cells through acting as ceRNA for miR-204-5p [61]. Decreased expression of TUG1 was demonstrated to inhibit trophoblast cell proliferation, migration, invasion and the formation of capillary-like networks and promote trophoblast cell apoptosis via epigenetic suppression of RND3 [40]. There is controversy regarding the expression of TUG1 in PE that could be explained based on the difference in sample nature (serum, placenta, or trophoblast cell lines), regulatory mechanisms, different normalization controls, and confounding factors. However, in this study, the remarked correlations of serum TUG1 level with albuminuria and ultrasound data in LOPE patients bolsters its relation to PE progression.

In general, the observed discrepancies of the present results with the literature could be due to different samples (circulation vs placental tissue or cell lines), different sampling methods, sample processing, time of sampling, normalization controls, and sample size. Nevertheless, we demonstrated that circulating H19, NEAT1, miR-29b, and SLC3A1 levels could be clinically useful in terms of their diagnostic potential and correlations with maternal and fetal parameters in EOPE and LOPE. These markers could be also therapeutically targeted either to prevent PE in high-risk pregnant women or to reduce PE progression.

This study is limited by several concerns. First, although we increased the initial sample size to reach a sufficient power, subgrouping of PE patients might decrease the statistical power. Despite recruiting the participants from two different hospitals to reduce the selection bias, not all cases were eligible as we implemented precise criteria for inclusion and exclusion, thus we weren't able to further enlarge the sample size in the fixed period of the study. Second, we couldn't get samples prior the onset of PE as in many cases PE is silent and is discovered by routine blood pressure and urine testing during prenatal visits. Third, the study is cross-sectional and we didn't make a longitudinal follow up between the progressions of mild to severe PE which may limit the certitude of PE prognosis. Fourth, we didn't confirm the relationship between studied lncRNAs/miRNA/target gene axis in trophoblast cell lines; however, their interactions were experimentally validated by previous studies. Thereby, we urge additional larger-scale longitudinal studies to confirm the current findings *in vivo* and *in vitro*. Nevertheless, our data lay the groundwork for identifying new clinical tools and therapeutic targets for implementation in individualized testing helped with the vast availability of technical facilities for gene expression assays.

## 5 Conclusion

Our study advocates serum H19, NEAT1, miR-29b, and SLC3A1 as new biomarkers in the diagnosis of PE, with superior diagnostic accuracy for H19 and miR-29b. Peculiarly, serum H19 and miR-29b may be associated with EOPE, whereas miR-29b is associated with severe PE, suggesting new markers for predicting PE onset and severity. This study also configures SLC3A1 expression as a novel potential serum biomarker of PE that correlates with ultrasound data in overall PE, EOPE, and LOPE patients. Serum TUG1, H19, NEAT1, and miR-29b correlate with clinical and ultrasound data of PE patients and could have utility in clinical practice. Our data widen the epigenetic landscape of PE and accentuate the progress made in the discovery of new biomarkers for the early detection of PE, and also spotlight on new therapeutic targets to expedite the future work in this field.

## Ethics approval

The ethical committee of Faculty of Pharmacy, Cairo University, Cairo, Egypt, approved all experiments (Permit number: BC3130). A full compliance with the guidelines and regulations in the Helsinki Declaration was applied in all methods.

## Consent for publication

Not applicable.

## Data availability

All data generated or analyzed during this study are included in this manuscript, tables, figures, and supplementary information.

## Funding

This current work was funded by 10.13039/501100006261Taif University, Saudi Arabia, Project number (TU-DSPP-2024-116).

## CRediT authorship contribution statement

**Mahmoud A. Senousy:** Writing – original draft, Visualization, Software, Methodology, Investigation, Formal analysis, Data curation, Conceptualization. **Olfat G. Shaker:** Resources, Methodology, Investigation, Conceptualization. **Ahmed H.Z. Elmaasrawy:** Writing – review & editing, Methodology, Investigation. **Ahmed M. Ashour:** Writing – review & editing, Funding acquisition. **Shuruq E. Alsufyani:** Writing – review & editing, Funding acquisition. **Hany H. Arab:** Writing – review & editing, Software, Methodology. **Ghada Ayeldeen:** Resources, Methodology, Investigation.

## Declaration of competing interest

The authors declare that they have no known competing financial interests or personal relationships that could have appeared to influence the work reported in this paper.
