# Supplementary material for: Serum lncRNAs TUG1, H19, and NEAT1 and their target miR-29b/SLC3A1 axis as possible biomarkers of preeclampsia: Potential clinical insights
Source: Noncoding RNA Res. 2024 Jun 8;9(4):995–1008. doi: 10.1016/j.ncrna.2024.06.007 (PMC11254728; doi:10.1016/j.ncrna.2024.06.007)
Supplement: Multimedia component 1 [file mmc1.docx]

**Supplementary data**

**Supplementary Table S1. PE-related non-coding RNAs according to LncRNADisease v2.0**

| **Database ID** | **Symbol** | **Category** | **SPecies** | **Disease Name** | **Detection Method** | **Score** | **Causality** |
| --- | --- | --- | --- | --- | --- | --- | --- |
| LDA0007982 | TUG1 | lncRNA | Homo saPiens | Pre-eclamPsia | ExPerimental/Predicted | 0.9580 | Yes |
| LDA0006028 | lnc-DC | lncRNA | Homo saPiens | Pre-eclamPsia | ExPerimental | 0.9526 | Yes |
| LDA0007979 | HOTAIR | lncRNA | Homo saPiens | Pre-eclamPsia | ExPerimental/Predicted | 0.9070 | Yes |
| LDA0007980 | MALAT1 | lncRNA | Homo saPiens | Pre-eclamPsia | ExPerimental/Predicted | 0.9070 | Yes |
| LDA0007981 | SPRY4-IT1 | lncRNA | Homo saPiens | Pre-eclamPsia | ExPerimental/Predicted | 0.9070 | Yes |
| LDA0006026 | CEACAMP8 | lncRNA | Homo saPiens | Pre-eclamPsia | ExPerimental | 0.8785 | Unknown |
| LDA0006027 | FLT1P1 | lncRNA | Homo saPiens | Pre-eclamPsia | ExPerimental | 0.8785 | Unknown |
| LDA0006030 | YWHAEP7 | lncRNA | Homo saPiens | Pre-eclamPsia | ExPerimental | 0.8785 | Unknown |
| LDA0007978 | H19 | lncRNA | Homo saPiens | Pre-eclamPsia | ExPerimental/Predicted | 0.7802 | Unknown |
| LDA0006029 | Uc.187 | lncRNA | Homo saPiens | Pre-eclamPsia | ExPerimental | 0.7311 | Yes |
| LDA0179170 | hsa_circ_101222 | circRNA | Homo saPiens | Pre-eclamPsia | ExPerimental | 0.7311 | Unknown |
| LDA0179171 | hsa_circRNA_100782 | circRNA | Homo saPiens | Pre-eclamPsia | ExPerimental | 0.7311 | Unknown |
| LDA0179172 | hsa_circRNA_102682 | circRNA | Homo saPiens | Pre-eclamPsia | ExPerimental | 0.7311 | Unknown |
| LDA0179173 | hsa_circRNA_104820 | circRNA | Homo saPiens | Pre-eclamPsia | ExPerimental | 0.7311 | Unknown |
| LDA0126371 | AC091891.2 | lncRNA | Homo saPiens | Pre-eclamPsia | Predicted | 0.2202 | Not Available |

**Supplementary Table S2. Predicted interactions with miR-29b according to the miRcode 11 data base.**

| **microRNA family** | **Seed Position** | **Seed**  **tyPe** | **TranscriPt**  **region** | **RePeat** | **Conservation** | | |
| --- | --- | --- | --- | --- | --- | --- | --- |
|  |  |  |  |  | **Primates** | **Mammals** | **Other vert.** |
| **ENSG00000130600.9 (H19)** | | | | | | | |
| miR-29abcd | [**chr11:2017218**](http://genome.ucsc.edu/cgi-bin/hgTracks?db=hg19&wgEncodeGencodeSuper=show&wgEncodeGencodeV12ViewGenes=full&wgEncodeGencodeBasicV12_sel=0&wgEncodeGencodeCompV12_sel=1&wgEncodeGencodePseudoGeneV12_sel=1&position=chr11:2017169-2017268&hgt.customText=http://www.mircode.org/mircode/gencode_mirsites_highconsfamilies.bed) | 8-mer | ncRNA | no | 56 % | 61 % | 0 % |
| **ENSG00000253352.3 (TUG1)** | | | | | | | |
| miR-29abcd | [**chr22:31366718**](http://genome.ucsc.edu/cgi-bin/hgTracks?db=hg19&wgEncodeGencodeSuper=show&wgEncodeGencodeV12ViewGenes=full&wgEncodeGencodeBasicV12_sel=0&wgEncodeGencodeCompV12_sel=1&wgEncodeGencodePseudoGeneV12_sel=1&position=chr22:31366669-31366768&hgt.customText=http://www.mircode.org/mircode/gencode_mirsites_highconsfamilies.bed) | 7-mer-m8 | ncRNA | no | 89 % | 70 % | 0 % |
| miR-29abcd | [**chr22:31367104**](http://genome.ucsc.edu/cgi-bin/hgTracks?db=hg19&wgEncodeGencodeSuper=show&wgEncodeGencodeV12ViewGenes=full&wgEncodeGencodeBasicV12_sel=0&wgEncodeGencodeCompV12_sel=1&wgEncodeGencodePseudoGeneV12_sel=1&position=chr22:31367055-31367154&hgt.customText=http://www.mircode.org/mircode/gencode_mirsites_highconsfamilies.bed) | 7-mer-m8 | ncRNA | no | 89 % | 78 % | 0 % |
| [**ENSG00000245532.2**](http://genome.ucsc.edu/cgi-bin/hgTracks?db=hg19&wgEncodeGencodeSuper=show&wgEncodeGencodeV12ViewGenes=full&wgEncodeGencodeBasicV12_sel=0&wgEncodeGencodeCompV12_sel=1&wgEncodeGencodePseudoGeneV12_sel=1&position=chr11:65190244-65213011&hgt.customText=http://www.mircode.org/mircode/gencode_mirsites_highconsfamilies.bed)**(NEAT1) (lncRNA, intergenic)** | | | | | | | |
| miR-29abcd | [**chr11:65195335**](http://genome.ucsc.edu/cgi-bin/hgTracks?db=hg19&wgEncodeGencodeSuper=show&wgEncodeGencodeV12ViewGenes=full&wgEncodeGencodeBasicV12_sel=0&wgEncodeGencodeCompV12_sel=1&wgEncodeGencodePseudoGeneV12_sel=1&position=chr11:65195286-65195385&hgt.customText=http://www.mircode.org/mircode/gencode_mirsites_highconsfamilies.bed) | 7-mer-m8 | ncRNA | no | 44 % | 4 % | 0 % |
| miR-29abcd | [**chr11:65198799**](http://genome.ucsc.edu/cgi-bin/hgTracks?db=hg19&wgEncodeGencodeSuper=show&wgEncodeGencodeV12ViewGenes=full&wgEncodeGencodeBasicV12_sel=0&wgEncodeGencodeCompV12_sel=1&wgEncodeGencodePseudoGeneV12_sel=1&position=chr11:65198750-65198849&hgt.customText=http://www.mircode.org/mircode/gencode_mirsites_highconsfamilies.bed) | 8-mer | ncRNA | yes | 44 % | 4 % | 0 % |
| [**ENSG00000138079.8**](http://genome.ucsc.edu/cgi-bin/hgTracks?db=hg19&wgEncodeGencodeSuper=show&wgEncodeGencodeV12ViewGenes=full&wgEncodeGencodeBasicV12_sel=0&wgEncodeGencodeCompV12_sel=1&wgEncodeGencodePseudoGeneV12_sel=1&position=chr2:44502598-44548633&hgt.customText=http://www.mircode.org/mircode/gencode_mirsites_highconsfamilies.bed)**(SLC3A1) (coding)** | | | | | | | |
| miR-29abcd | [**chr2:44502974**](http://genome.ucsc.edu/cgi-bin/hgTracks?db=hg19&wgEncodeGencodeSuper=show&wgEncodeGencodeV12ViewGenes=full&wgEncodeGencodeBasicV12_sel=0&wgEncodeGencodeCompV12_sel=1&wgEncodeGencodePseudoGeneV12_sel=1&position=chr2:44502925-44503024&hgt.customText=http://www.mircode.org/mircode/gencode_mirsites_highconsfamilies.bed) | 7-mer-m8 | CDS | no | 67 % | 9 % | 23 % |
| miR-29abcd | [**chr2:44531156**](http://genome.ucsc.edu/cgi-bin/hgTracks?db=hg19&wgEncodeGencodeSuper=show&wgEncodeGencodeV12ViewGenes=full&wgEncodeGencodeBasicV12_sel=0&wgEncodeGencodeCompV12_sel=1&wgEncodeGencodePseudoGeneV12_sel=1&position=chr2:44531107-44531206&hgt.customText=http://www.mircode.org/mircode/gencode_mirsites_highconsfamilies.bed) | 7-mer-A1 | 5PUTR | no | 56 % | 0 % | 0 % |
| miR-29abcd | [**chr2:44548540**](http://genome.ucsc.edu/cgi-bin/hgTracks?db=hg19&wgEncodeGencodeSuper=show&wgEncodeGencodeV12ViewGenes=full&wgEncodeGencodeBasicV12_sel=0&wgEncodeGencodeCompV12_sel=1&wgEncodeGencodePseudoGeneV12_sel=1&position=chr2:44548491-44548590&hgt.customText=http://www.mircode.org/mircode/gencode_mirsites_highconsfamilies.bed) | 7-mer-m8 | 3PUTR | no | 89 % | 57 % | 0 % |

Interactions are based on the GENCODE.

**Supplementary Table S3. Interaction of miR-29b-3p and human SLC3A1 according to TargetScan 7.0**

| **Predicted consequential Pairing of target region (toP) and miRNA (bottom)** | **Site tyPe** | **Context++ score** | **Context++ score Percentile** | **Weighted context++ score** | **Conserved branch length** | **P_CT_** |
| --- | --- | --- | --- | --- | --- | --- |
| Position 755-761 of SLC3A1 3' UTR  [hsa-miR-29b-3P](http://www.mirbase.org/cgi-bin/mirna_entry.pl?acc=hsa-miR-29b-3p) | 5'  ...GAUCCUCGAAAACACUGGUGCUG...                       \|\|\|\|\|\|\|  3'     UUGUGACUAAAGUUUACCACGAU | 7mer-m8 | -0.19 | 82 | -0.19 | 2.950 |

The SLC3A1 transcriPt shown is ENST00000260649.6 3' UTR length: 855

**Supplementary Table S4. Correlation study in healthy Pregnancies**

|  |  | H19 | TUG1 | NEAT1 | miR-29b | SLC3A1 |
| --- | --- | --- | --- | --- | --- | --- |
| H19 | r | - | -0.142 | -0.015 | 0.062 | 0.289 |
|  | P | - | 0.549 | 0.949 | 0.795 | 0.090 |
| TUG1 | r | -0.142 | - | -0.176 | -0.083 | -0.034 |
|  | P | 0.549 | - | 0.458 | 0.727 | 0.887 |
| NEAT1 | r | -0.015 | -0.176 | - | -0.189 | 0.128 |
|  | P | 0.949 | 0.458 | - | 0.423 | 0.590 |
| miR-29b | r | 0.062 | -0.083 | -0.189 | - | -0.160 |
|  | P | 0.794 | 0.727 | 0.423 | - | 0.500 |
| SLC3A1 | r | 0.289 | -0.034 | 0.128 | -0.160 | - |
|  | P | 0.09 | 0.887 | 0.590 | 0.500 | - |
| Age | r | -0.012 | -0.170 | 0.196 | 0.002 | 0.002 |
|  | P | 0.961 | 0.473 | 0.406 | 0.995 | 0.992 |
| BMI | r | 0.204 | -0.104 | -0.126 | -0.146 | 0.230 |
|  | P | 0.306 | 0.663 | 0.596 | 0.538 | 0.209 |
| Smoking | r | -0.010 | -0.010 | 0.111 | 0.081 | 0.205 |
|  | P | 0.966 | 0.966 | 0.641 | 0.735 | 0.287 |
| SBP | r | 0.077 | -0.170 | -0.173 | 0.158 | 0.170 |
|  | P | 0.745 | 0.473 | 0.313 | 0.504 | 0.473 |
| DBP | r | **0.529** | -0.203 | 0.057 | -0.101 | 0.208 |
|  | P | **0.016** | 0.309 | 0.809 | 0.672 | 0.253 |
| MAP | r | 0.204 | -0.204 | -0.179 | 0.083 | 0.205 |
|  | P | 0.301 | 0.280 | 0.450 | 0.728 | 0.286 |
| GA | r | 0.199 | -0.056 | 0.230 | -0.159 | 0.147 |
|  | P | 0.39 | 0.813 | 0.144 | 0.502 | 0.535 |
| Platelet count | r | -0.128 | -0.027 | 0.148 | -0.178 | 0.219 |
|  | P | 0.589 | 0.909 | 0.534 | 0.452 | 0.254 |
| CRP | r | 0.246 | -0.098 | 0.193 | 0.206 | 0.149 |
|  | P | 0.114 | 0.679 | 0.415 | 0.316 | 0.529 |
| IUGR | r | -0.073 | 0.158 | -0.134 | 0.097 | -0.206 |
|  | P | 0.760 | 0.506 | 0.572 | 0.682 | 0.382 |
| DoPPler | r | -0.058 | 0.247 | -0.209 | -0.073 | 0.000 |
|  | P | 0.808 | 0.134 | 0.213 | 0.761 | 1.000 |
| AF | r | -0.241 | -0.061 | 0.208 | 0.012 | -0.255 |
|  | P | 0.142 | 0.799 | 0.379 | 0.959 | 0.178 |
| MOD | r | -0.038 | 0.184 | 0.148 | -0.243 | 0.241 |
|  | P | 0.874 | 0.225 | 0.292 | 0.139 | 0.142 |
| FBW | r | 0.199 | 0.002 | 0.178 | -0.208 | 0.243 |
|  | P | 0.400 | 0.994 | 0.452 | 0.186 | 0.139 |

Correlations were done using Spearman correlation in the control healthy pregnancies group (n=78). Bold indicates P<0.05: statistically significant. AF, amniotic fluid; BMI, body mass index; CRP, C-reactive Protein; DBP, diastolic blood Pressure; FBW, fetal birth weight; GA, gestational age; IUGR, intrauterine growth restriction; MAP, mean arterial Pressure; MOD, mode of delivery; SBP, systolic blood Pressure.
